# Exercise and dietary intervention ameliorate high-fat diet-induced NAFLD and liver aging by inducing lipophagy

**DOI:** 10.1016/j.redox.2020.101635

**Published:** 2020-07-07

**Authors:** Yu Gao, Wei Zhang, Li-Qin Zeng, Hua Bai, Jia Li, Jian Zhou, Geng-Yao Zhou, Cong-Wen Fang, Feng Wang, Xu-Jun Qin

**Affiliations:** a Department of Nutrition and Food Hygiene, School of Preventive Medicine, The Fourth Military Medical University, Xi'an, 710032, China; b Department of Cardiovascular Diseases, Tangdu Hospital, The Fourth Military Medical University, Xi'an, 710032, China; c Department of Ultrasound, Xijing Hospital, The Fourth Military Medical University, Xi'an, 710032, China; d Department of Toxicology, School of Preventive Medicine, The Fourth Military Medical University, Xi'an, 710032, China; e Department of Aerospace Medicine, The Fourth Military Medical University, Xi'an, 710032, China

**Keywords:** Nonalcoholic fatty liver disease (NAFLD), Lipophagy, FGF21, Exercise, Aging

## Abstract

Exercise and dietary intervention are currently available strategies to treat nonalcoholic fatty liver disease (NAFLD), while the underlying mechanism remains controversial. Emerging evidence shows that lipophagy is involved in the inhibition of the lipid droplets accumulation. However, it is still unclear if exercise and dietary intervention improve NAFLD through regulating lipophagy, and how exercise of skeletal muscle can modulate lipid metabolism in liver. Moreover, NAFLD is associated with aging, and little is known about the effect of lipid accumulation on aging process. Here in vivo and in vitro models, we found that exercise and dietary intervention reduced lipid droplets formation, decreased hepatic triglyceride in the liver induced by high-fat diet. Exercise and dietary intervention enhanced the lipophagy by activating AMPK/ULK1 and inhibiting Akt/mTOR/ULK1 pathways respectively. Furthermore, exercise stimulated FGF21 production in the muscle, followed by secretion to the circulation to promote the lipophagy in the liver via an AMPK-dependent pathway. Importantly, for the first time, we demonstrated that lipid accumulation exacerbated liver aging, which was ameliorated by exercise and dietary intervention through inducing lipophagy. Our findings suggested a new mechanism of exercise and dietary intervention to improve NAFLD through promoting lipophagy. The study also provided evidence to support that muscle exercise is beneficial to other metabolic organs such as liver. The FGF21-mediated AMPK dependent lipophagy might be a potential drug target for NAFLD and aging caused by lipid metabolic dysfunction.

## Graphical abstract

**Figure:**
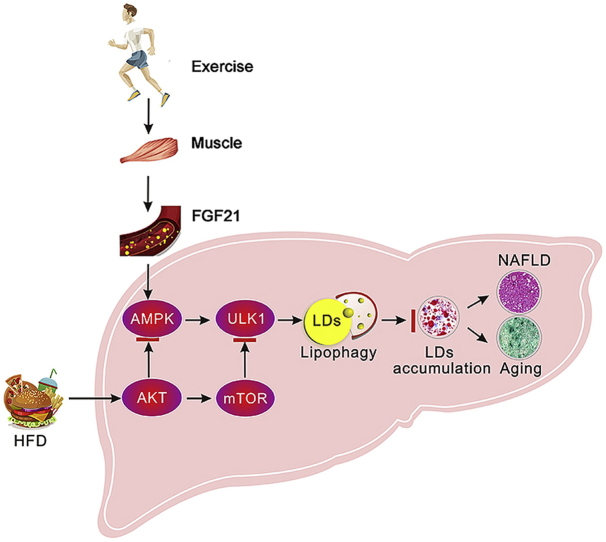

## 1 Introduction

Nonalcoholic fatty liver disease (NAFLD) is a progressive liver disease with a histological spectrum ranging from steatosis to fibrosis and cirrhosis. It is characterized as excessive accumulation of triglyceride (TG) and cholesterol in lipid droplets (LDs) in hepatocytes [1]. Currently, NAFLD is considered as one of the most common causes of chronic liver diseases which leads to 1.2 million deaths annually and rises to the 8th most common cause of death worldwide [2]. However, there is currently no approved drugs for the treatment of NAFLD. Therefore, understanding of the precise mechanisms underlying the onset of NAFLD is critical for development of successful therapeutic strategies.

In the past decade, accumulating evidence has shown that autophagy is involved in the hepatic lipid metabolism and plays a crucial role in the NAFLD pathogenesis [3,4]. Inhibition of autophagy with 3-methyladenine (3-MA) or atg5 siRNA knockdown in a rat hepatocyte cell line increased TG levels as well as the number and size of LDs, suggesting the autophagy induction may be an effective therapy for NAFLD [5]. LDs are large stores of neutral lipid esters, triglycerides, and cholesteryl esters in the cells. The metabolism of LDs regulates the metabolic state of the liver. Excess LDs accumulation in the hepatocytes is the first step of the NAFLD pathogenesis. Hence, suppressing the LDs accumulation would be the important strategy for NAFLD treatment. Autophagy involving in LDs lipolysis is termed as “lipophagy”. As a selective autophagy, lipophagy specifically targets and clears excess lipid in the liver. Mutation of the peptide motif in PLIN2, the major perilipin on the surface of the LDs involved in the lipophagy, which can be recognized by LC3, resulted in LDs accumulation in the liver [6]. Pharmacological inhibition of lipophagy significantly decreased the lipolytic breakdown of TG and cholesterol from LDs, leading to increased liver steatosis after a lipid challenge [4,7]. However, the mechanism of the lipophagy in the pathogenesis of NAFLD remained controversial [8].

Caloric restriction (CR) is the most common therapy for the NAFLD as well as obesity. However, how dietary intervention regulates lipophagy and whether the lipophagy regulation could be therapeutic target for NAFLD are still unclear. In addition to dietary intervention, numerous studies have found that exercise could improve glucose-lipid metabolism disorders [9,10]. However, the mechanism of how exercise reduces intrahepatic lipids independent of energy expenditure and how muscle contraction regulates liver lipid metabolism remain unknown. In addition to its important role in clearance of circulating glucose and fatty acids, skeletal muscle was demonstrated to be a secretory organ responsible for the production of several hundreds of peptides classified as ‘myokines’. These muscle-derived secretory factors may elicit endocrine effects on other organs. For example, IL-6, a well-described myokine secreted from myocytes, could stimulate lipolysis in adipose tissue. Likewise, it is believed that muscle might secrete some myokines to regulate liver metabolism [11,12].

For the past few years, fibroblast growth factor 21 (FGF21) is considered as a major insulin and exercise responsive myokine, which mediates some of the metabolic benefits of exercise [[13], [14], [15]]. Although the canonical role of FGF21 is associated with the upregulation of regulators of hepatic fatty acid oxidation and downregulation of regulators of hepatic lipogenesis, other novel mechanisms have been suggested for the function of FGF21 in regulating liver fat [16]. Recently, it was reported that FGF21 administration activated the AMPK, a central regulator of the autophagy pathway, promoted mitochondrial biogenesis and enhanced mitochondrial oxidative function in cultured rodent and human adipocytes [17]. Together, we hypothesized that muscle exercise may improve NAFLD by producing FGF21, which may induce lipophagy in the liver.

In the present study, we found that both exercise and dietary intervention induced lipophagy in the liver through multiple molecular pathways to improve the NAFLD. Furthermore, combination of these treatments would be more effective in treating NAFLD. We also found that exercise promoted FGF21 secretion from muscle, which mediated the lipophagy through an AMPK dependent pathway in the liver thus ameliorated NAFLD. More importantly, for the first time, we found that lipid accumulation exacerbated liver aging. Exercise and dietary intervention could attenuate liver aging through inducing lipophagy.

## 2 Materials and methods

### 2.1 Animal experiments

Healthy adult male Sprague-Dawly (SD) rats with a body weight ranging from 180 to 200 g were obtained from the Laboratory Animal Unit, the Fourth Military Medical University (Xi’An, China). Rats were maintained and cared in compliance with the requirements of the Fourth Military Medical University. All experiments were approved by the Fourth Military Medical University Animal Ethics Committee. At 6 weeks of age, the high-fat diet (HFD) group was fed a HFD (45 kcal% fat, Cat. No. D12451, Research Diets; New Brunswick, NJ, USA) ad libitum for 12 weeks to induce obesity. After the first 12 weeks of HFD, rats were randomly subdivided into HFD without exercise (n = 8), HFD with exercise (n = 8), dietary change back to chow-diet without exercise (n = 8), and dietary change back to chow with exercise (n = 8) groups.

### 2.2 Exercise training protocol

All rats were acclimated on a motor-driven treadmill (ZH-PT, Zhenghua Biological Instruments, Anhui, China) for five consecutive days as pre-exercise training for adaptation (detailed in Supplementary Table 1). Following a 4-day rest period, rats began the treadmill running at 20 m/min for 60min per day, and 5 days per week for the final 8 weeks.

### 2.3 Cell culture and FFA treatment

HepG2 cells were maintained in Dulbecco's modified Eagle's medium (DMEM) (Cat. No. 10566016, Gibco), and WI-38 cells were in minimum Eagle's medium, containing 10% fetal bovine serum (FBS) (Cat. No. 10100154, Gibco) and 1% penicillin/streptomycin (Cat. No. 15140122, Gibco) (defined as full medium in this study) in a 5% CO_2_ atmosphere at 37 °C. The mixture of oleate (Cat. No. O3880, Sigma) and palmitate (Cat. No. P9767, Sigma) was used as FFA to induce the lipid accumulation in the cells [18]. In brief, 0.5 M BSA (Cat. No. 03117405001, Roche applied sciences) solution was prepared by dissolving it in 150 mM NaCl solution. Oleate and palmitate were dissolved in 0.5 M BSA to make a 4 mM total FFA mixture (Oleate: palmitate 2:1 M ration), and the products were filtered. The stock solution was conveniently diluted in culture medium (1:10) to obtain the 400 μM final concentration. After the cells were treated with FFA for 10 days, the FGF21 (1 μg/ml) as well as AMPK inhibitor-Dorsomorphin (1 μmol/L) were added to the intervention groups to co-treat the cells with FFA for additional 4 days.

### 2.4 Quantification of TG

Hepatic TG level was measured as described before [19]. Animals were sacrificed and the livers were frozen in liquid nitrogen immediately, then stored at −80 °C until processed. Frozen livers were weighted and homogenized in lysis buffer (140 mM NaCl, 50 mM Tris, 0.1% Triton-X) using a tissuelyser (Qiagen). For the culture cells, when cells were 90% confluent, they were treated with the same lysis buffer and centrifuged to get the lysates. Liver homogenates or cell lysates were then incubated with 1% deoxycholate at 37 °C for 10 min, and triglycerides were measured by an enzymatic method using Infinity Triglyceride Reagent (Cat. No. TR22421, Thermo Fisher Scientific). The results were normalized by the protein concentrations.

### 2.5 Histological analysis

For H&E staining, liver tissues were fixed in 4% paraformaldehyde, embedded in paraffin, and cut into 4-mm sections subsequent to H&E staining. Oil Red O staining was performed as described [20]. The frozen livers were sectioned at 7 μm thickness with a Leica cryomicrotome (Leica Microsystems) after fixation in 4% paraformaldehyde for 10 min. Following three washes with distilled water, the slides were placed in absolute propylene glycol for 5 min. Slides were then incubated in pre-warmed saturated 60% isopropanol Oil Red O solution for 10 min in an oven at 60 °C. Slides were rinsed twice with distilled water, mounted with aqueous mounting media and cover slipped. Sections were digitalized using an Olympus BX-51 light microscope.

### 2.6 BODIPY staining

HepG2 cells were seed in confocal dishes to 70% confluence, and then the BODIPY staining were carried out as described in the introduction of the product. The cells were incubated with 1 μg/ml BODIPY 493/503 (Cat. No. D3922, Invitrogen) for 3 h. To image BODIPY 493/503 staining, the 488 nm laser line was used and signals were collected with a long pass filter 505 nm. Images were collected with a confocal laser scanning microscope (LSM800, Carl Zeiss), using a 63 × oil immersion objective.

### 2.7 Quantitative RT-PCR

Total RNA was isolated from tissues by using TRIzol (Cat No: 15596026, Thermo Fisher Scientific) and quantified with Nanodrop. Then 1 μg of RNA were reverse-transcribed with iScript™ cDNA Synthesis Kit (Cat. No. 1708890, BioRad). Quantitative RT-PCR analysis was performed with the Power SYBR™ Green PCR Master Mix (Cat. No. 4367659, Life Technologies) and the CFX Connect Realtime System (BioRad). The following PCR primers were used: rat FGF21, 5′-AGGCCTGCAGTTTCAGAGAG-3‘ (forward) and 5′-GATCCTGGGAGTCCTTCTGG-3‘ (reverse); rat p16, 5′-GGTTTTCTTGGTGCAGTTCC-3‘ (forward) and 5′-GATCCTCTCTGGCCTCAACA-3‘ (reverse); rat p27, 5′-TTGGGTCTCAGGCAAACTCT-3‘ (forward) and TCTGACGAGTCAGGCATTTG-3‘lsquo; (reverse); rat actin, 5′ AGATCCTGACCGAGCGTGGC-3‘ (forward) and 5′-CCAGGGAGGAAGAGGATGCG-3‘ (reverse); human p16, 5′-GACCTGGCTGAGGAGCTG-3‘ (forward) and 5′-GCATGGTTACTGCCTCTGGT-3‘ (reverse); human p27, 5′-CATTTGGTGGACCCAAAGAC-3‘ (forward) and 5′-TTGCAGGTCGCTTCCTTATT-3‘ (reverse); human actin, 5′-GAGCGCGGCTACAGCTT-3‘ (forward) and 5′-TCCTTAATGTCACGCACGATTT-3‘ (reverse). Relative gene expression was obtained after normalization to actin expression. Fold differences in comparisons were expressed as relative mRNA levels using the 2^−ΔΔ*Ct*^ method [18].

### 2.8 Western blot analysis

The Western blot analysis was performed as described previously [21] using specific antibodies. *Anti*-LC3 antibody (Cat. No. NB100-2220) was from Novus Biologicals (Centennial, CO, USA). *Anti*-Atg7 antibody (Cat. No. 8558), Beclin-1 (Cat. No. 3495), Lamp1 (Cat. No. 3243), AMPK (Cat. No. 5832), *p*-AMPK (Thr172) (Cat. No. 2535), mTOR (Cat. No. 2972), *p*-mTOR (Ser 2448) (Cat. No. 2971), ULK1 (Cat. No. 8054), *p*-ULK1 (Ser555) (Cat. No. 5869), *p*-ULK1 (Ser757) (Cat. No. 14202), Akt (Cat. No. 9272), *p*-Akt (Ser473) (Cat. No. 4058) antibodies were from Cell Signaling Technology (Beverly, MA, USA). Atg5 (Cat. No. ab108327), Lamp2 (Cat. No. ab125068) and p62 (Cat. No. ab109012) antibodies were from Abcam (Abcam, Cambridge, UK). *p*-AMPK (Ser487) (Cat. No. BS4010), Tubulin (Cat. No. BS1699) antibodies were from Bioworld Technology (Louis Park, MN, USA). RIPA lysis buffer was used to prepare rat tissue and cell lysates. Lysosomes were isolated with the kit from BestBio (Cat. No.BB3603, Shanghai, China) according to the manufacturer's protocol, and lysed with RIPA lysis buffer to prepare the lysosome proteins. 10–20 μg protein was loaded and separated on SDS-PAGE gels. Fractionated proteins were then transferred to nitrocellulose membranes, blocked in 5% nonfat milk for 2 h, and probed overnight with primary antibodies. Immunoblots were washed three times (5 min each) with TBS containing 0.1% Tween 20 and then incubated with horseradish peroxidase conjugated secondary antibody for 2 h. Blots were washed four times (5 min each) with TBS containing 0.1% Tween 20, developed in enhanced chemiluminescent reagent (Cat. No. WBKLS0500, MilliporeSigma), and visualized with an image analyzer Quantity One System (Bio-Rad).

### 2.9 Measurements of serum FGF21

Rat FGF21 enzyme-linked immunosorbent assay (ELISA) kit was obtained from Cusabio Biotech (Cat. No. CSB-EL008627RA, Cusabio Biotech, Wuhan, China). For the measurement of FGF21, 100 μl serum or tissue samples, calibrators, and quality controls were added to 96-well microtiter plates coated with anti-rat FGF21 antibody. The assay was conducted according to the manufacturer's protocol. The results in tissue were normalized by the protein concentrations.

### 2.10 Autophagic flux quantification

Autophagic flux in RFP-GFP-LC3 adeno virus infected HepG2 cells were performed using an imaging-based assay as previously reported [22,23]. Briefly, 30–60 cells for each condition were quantified. The red only was considered as autolysosome, while yellow was early autophagosome alone (Red and Green = yellow). Because of the acidic pH, the GFP fluorescence was diminished while RFP still remains stable. The conversion of yellow puncta to red puncta provided a readout for autophagic flux. The puncta in cells were analyzed with a confocal laser scanning microscope (LSM800, Carl Zeiss), using a 63 × oil immersion objective. The yellow puncta and red only puncta were quantified with the Image J program [23].

### 2.11 Determination of lipophagy levels

Lipophagy levels were measured as previously described [24]. HepG2 cells were infected with RFP-LC3 lentivirus and 1 μg/ml BODIPY 493/503 was added 3 h prior to imaging to visualize autophagosomes and LDs, respectively. The co-localization of RFP-LC3 with BODIPY 493/503 in cells were analyzed with a confocal laser scanning microscope (LSM800, Carl Zeiss), using a 63 × oil immersion objective.

### 2.12 SA-β-Gal measurement

For histochemical staining for SA-β-Gal, frozen sections were rehydrated three times, 5min each, with PBS. Sections were then immersed in β-galactosidase solution (1 mg/ml 5-bromo-4-chloro-3-indolyl-beta-gal (X-gal) in 5 mM potassium ferricyamide, 5 mM potassium ferrocyamide, 2 mM MgCl_2_ in PBS). After incubation in the dark at 37 °C for 16–18 h until beta-Gal staining become visible, sections were washed in PBS, mounted with aqueous mounting media and cover slipped. Images were collected with an Olympus BX-51 light microscope. For Cytochemical staining for SA-β-Gal, cells were fixed for 15 min in 4% paraformaldehyde (0.1 M phosphate buffer), then the staining was performed as the procedure described above. The SA-β-Gal activities in the liver or cells were determined with the β-Gal activity detection kit from BioLab (Cat. No.SK170-2, Beijing, China). The main procedures were according to the introduction of the kit.

### 2.13 Lipofuscin measurement

The lipofuscin in liver were measured according to the method as previously reported [25]. Tissues were homogenized in chloroform-methanol mixture (1:20, w/v). After centrifugation, collected the chloroform rich layer, and then mixed with methanol. An emission spectrum between 350 and 550 nm was obtained at an excitation wavelength of 350 nm using a TECAN 200 Pro plate reader. Lipofuscin concentrations were expressed as relative fluorescence intensity (RFI). Quinine sulfate at a concentration of 0.1 mg/1 in H_2_SO_4_ was used as a fluorescence standard. The lipofuscin in the cells were detected with the human lipofuscin enzyme-linked immunosorbent assay (ELISA) kit from JiangLai Bio (Cat. No.JL20055, Shanghai, China).

### 2.14 Malondialdehyde (MDA)

Liver tissues or cells were homogenized lysed with lysis buffer from Beyotime Biotechnology (Cat. No. P0013, Shanghai, China), and MDA was determined with a MDA detection kit from Beyotime Biotechnology (Cat. No. S0131) strictly following the protocol instructions provided with the kit.

### 2.15 Statistical analysis

Data are presented as means ± SEM. Intergroup comparisons were performed by two-way ANOVA and significance was accepted at *P* < 0.05.

## 3 Results

### 3.1 Exercise and dietary intervention (changing from HFD to regular chow) attenuated the HFD-induced liver steatosis

**Fig. 1:**
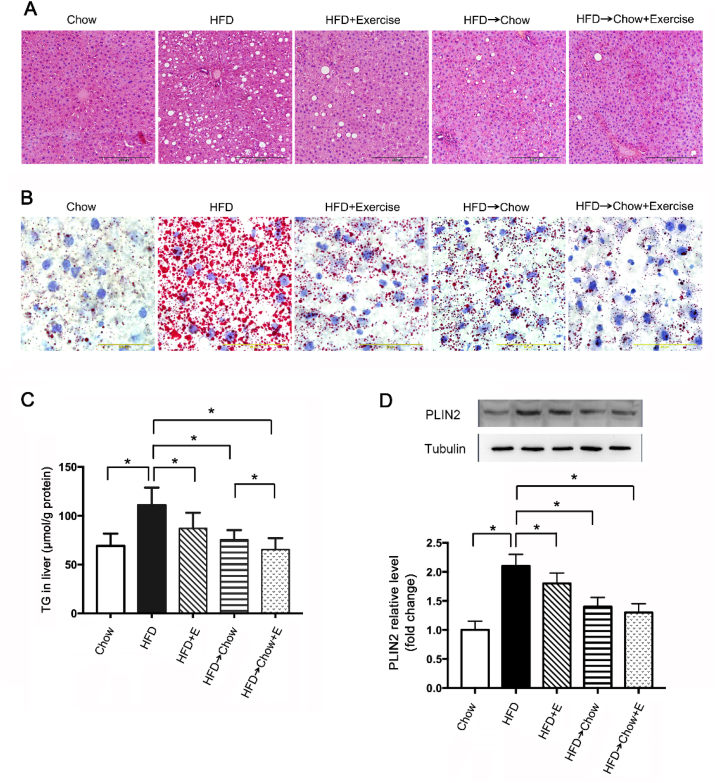
Exercise and dietary intervention protected against the HFD-induced liver steatosis. (A and B): Representative H&E staining and Oil Red O staining in the liver from rats on HFD with exercise and dietary intervention. Scale: 200 μm for A, Scale: 50 μm for B·(C and D) TG level and PLIN2 protein level in livers from rats on HFD with exercise and dietary intervention. Data are shown as the means ± SEM, n = 8 per group. Significance was designated by asterisks with *p < 0.05. (For interpretation of the references to colour in this figure legend, the reader is referred to the Web version of this article.)

Exercise and calorie restriction could improve the NAFLD by increasing energy expenditure, decreasing lipid overload as well as improving metabolic homeostasis [26]. In the present study, HFD induced a significant hepatic steatosis, indicated by higher lipid-laden white area present within cells in hematoxylin and eosin (H&E) stains (Fig. 1A) and Oil Red O staining (Fig. 1B). Both exercise and dietary intervention reduced the hepatic lipid content respectively. In order to accurately evaluate the effects of exercise and dietary intervention on the hepatic steatosis, we determined the TG level in liver and analyzed the interaction between the two interventions. As shown in Fig. 1C, exercise or dietary intervention alone significantly reduced the liver TG level (p=0.017, p < 0.001), and the combination of the two treatments was more effective (p < 0.001). However, there was no obvious interaction between the two interventions (p=0.131). The liver fat content was further confirmed by the lipid droplet marker PLIN2 level shown in Fig. 1D.

### 3.2 Exercise and dietary intervention induced autophagy as well as lipophagy in the liver via different pathways

**Fig. 2:**
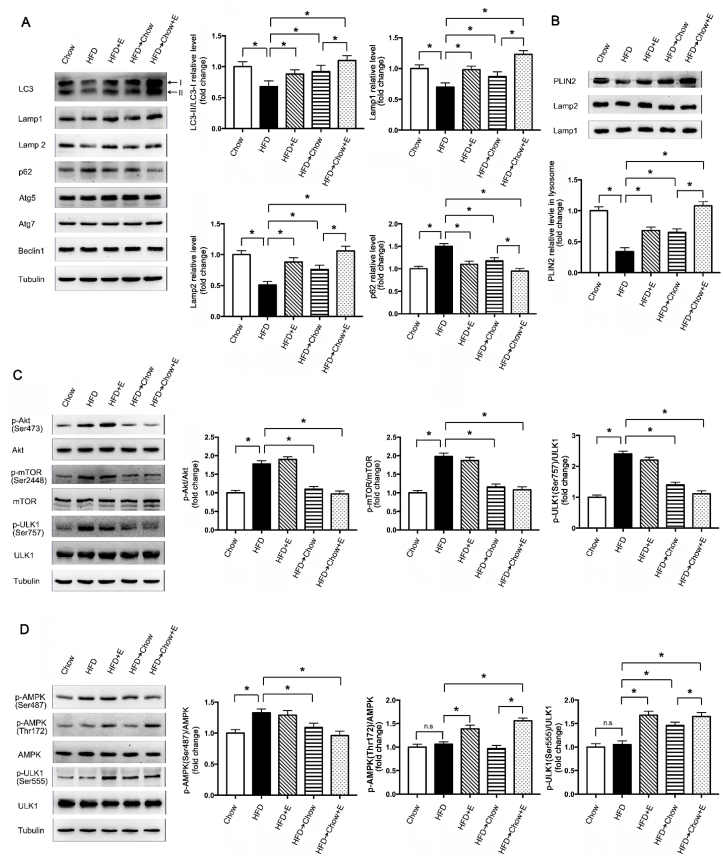
Exercise and dietary intervention improved the autophagy as well as lipophagy via different pathways. (A)Representative of autophagy-associated proteins immunoblotting and quantified data, (B) representative of lipophagy-associated protein PLIN2 immunoblotting in lysosome and quantified data, (C) representative of *p*-Akt (Ser473)/Akt total, p-mTORC1 (Ser2448)/mTORC1 total and *p*-ULK1 (Ser757)/ULK1 total immunoblotting and quantified data, (D) representative of *p*-AMPK (Ser487/Thr172)/AMPK total and *p*-ULK1 (Ser555)/ULK1 total immunoblotting and quantified data from rats on HFD with exercise and dietary intervention. Experiments were repeated three times. Data are shown as the means ± SEM. Significance was designated by asterisks with *p < 0.05.

As a catabolic process, autophagy is crucial for the maintenance of metabolic homeostasis by removing abnormal organelles, excess lipids, and protein aggregates in the liver. Many studies suggested that the defect of autophagy played an important role in the NAFLD [27]. In the present study, it was shown that HFD significantly decreased the protein levels of LC3II/LC3I, Lamp1 and Lamp2, and increased the level of p62, suggesting the autophagy in the liver was suppressed by HFD. Exercise and dietary intervention both markedly augmented the autophagy as illustrated by the reversal of the protein levels of LC3II/LC3I, Lamp1, Lamp2 as well as p62. The combination of these two treatments was more effective (Fig. 2A). Lipophagy, a type of selective autophagy, could specifically degrade the excess lipid to regulate lipid storage in the cell. During lipophagy, the autophagosome sequesters lipid droplet, then fuses with lysosome to degraded the cargo in the autolysosome. The lipid droplet coat protein PLIN2 level in the lysosome is a marker for lipophagy [28]. In the present study, exercise or dietary intervention could preserve PLIN2 in the lysosome which was inhibited by HFD, indicating lipophagy was induced in the liver by exercise or dietary intervention. Furthermore, combining the exercise with dietary intervention was more effective (Fig. 2B).

ULK1, an ATG1 homologue in mammals, is a key regulator in autophagy initiation. Two phosphorylation sites of ULK1 are associated with autophagy, Ser555 and Ser757. Phosphorylation of Ser555 induced autophagy, whereas the phosphorylation at Ser757 suppressed autophagy [29]. In the present study, HFD induced the increase of phosphorylation of ULK1 at Ser757, while exercise could significantly increase the phosphorylation at Ser555, suggested HFD suppressed autophagy through inducing ULK1 phosphorylation at Ser757, and exercise increased autophagy by the phosphorylation at Ser555 (Fig. 2C).

mTOR is a well-established autophagy negative regulator through phosphorylation of ULK1 at Ser757 [30]. The mTOR is a downstream target of Akt. In the present study, HFD resulted in a significant activation of Akt, and further increased mTOR phosphorylation at Ser2448, which can be reversed by dietary intervention, whereas exercise had no obvious effect on this pathway (Fig. 2C). AMPK is another upstream kinase which phosphorylates ULK1 at Ser555. Exercise significantly elevated the AMPK phosphorylation at Thr172, which is required for the activation of AMPK [31], while AMPK phosphorylation at Ser487, which suppresses AMPK activity [32], was increased moderately in HFD group, suggesting that AMPK activity was suppressed by HFD, whereas exercise could stimulate its activity (Fig. 2D). It has been reported that Akt can phosphorylate AMPK at Ser487 [33]. The increase of phosphorylation of AMPK at Ser487 with the activation of Akt also suggested that HFD may regulate AMPK through Akt pathway. Altogether, exercise and dietary intervention could regulate the same target, “lipophagy” by different pathways. Dietary intervention increased the lipophagy by inhibiting Akt/mTOR/ULK1 pathway, whereas exercise stimulated lipophagy by activating AMPK/ULK1 pathway.

### 3.3 Exercise increased FGF21 expression in the muscle and promoted its secretion to the circulation

**Fig. 3:**
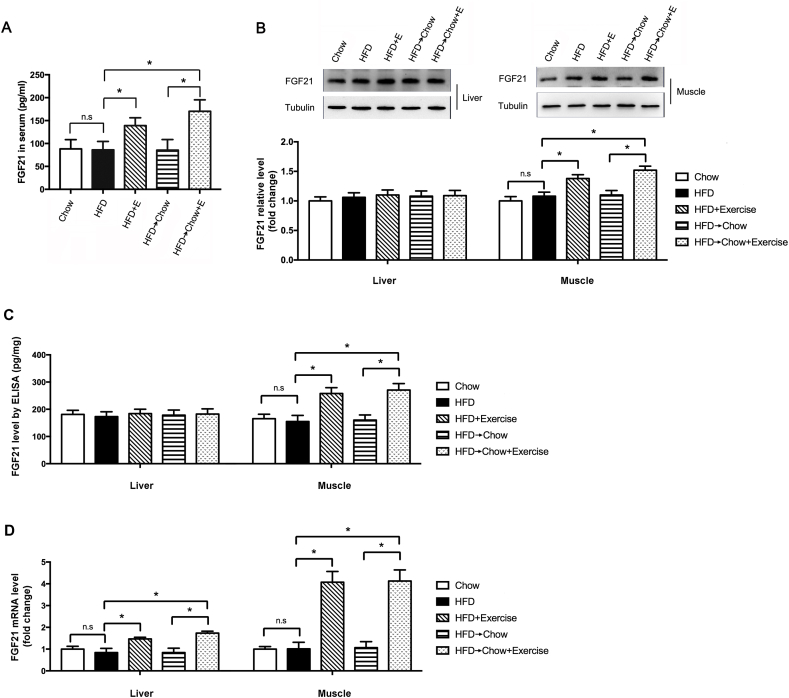
Exercise increased the FGF21 levels in serum and muscle. (A) FGF21 level in serum by ELISA. (B) FGF21 protein levels in liver and muscle by Western Blot. (C) FGF21 content in liver and muscle by ELISA. (D) FGF21 mRNA levels in liver and muscle by qPCR. Data are shown as the means ± SEM, n = 8 per group. Significance was designated by asterisks with *p < 0.05.

How exercise of muscles regulate liver lipophagy remains unknown. FGF21 is an important myokine secreted from muscle during exercise. Emerging evidence revealed that FGF21 can modulate metabolism through mediating the glucose disposal as well as energy expenditure. We found exercise significantly increased the FGF21 level in the serum (Fig. 3A), suggesting exercise may regulate liver metabolic homeostasis by inducing FGF21 production. It is reported that FGF21 was produced mostly in liver and muscle to regulate the metabolic status. To explore the source of FGF21 in serum during exercise in the present model, we evaluated the expression of FGF21 in liver and muscle. Data from the Western blot showed that exercise induced FGF21 significantly in muscle while there was no obvious change of FGF21 in liver (Fig. 3B). This was confirmed by the data of FGF21 by ELISA (Fig. 3C). To determine the FGF21 expression in these two tissues, we measured FGF21 mRNA levels in these two tissues. Exercise induced more than 4-fold of FGF21 mRNA in muscle, while only 0.7-fold increase in liver (Fig. 3D). All these data suggested that exercise promoted the FGF21 production in the muscle which was probably the main source of the elevated FGF21 in circulation. The muscle-derived FGF21 might be secreted to regulate liver metabolism.

### 3.4 FGF21 promoted the lipophagy and reduced the lipid accumulation through AMPK-dependent pathway

**Fig. 4:**
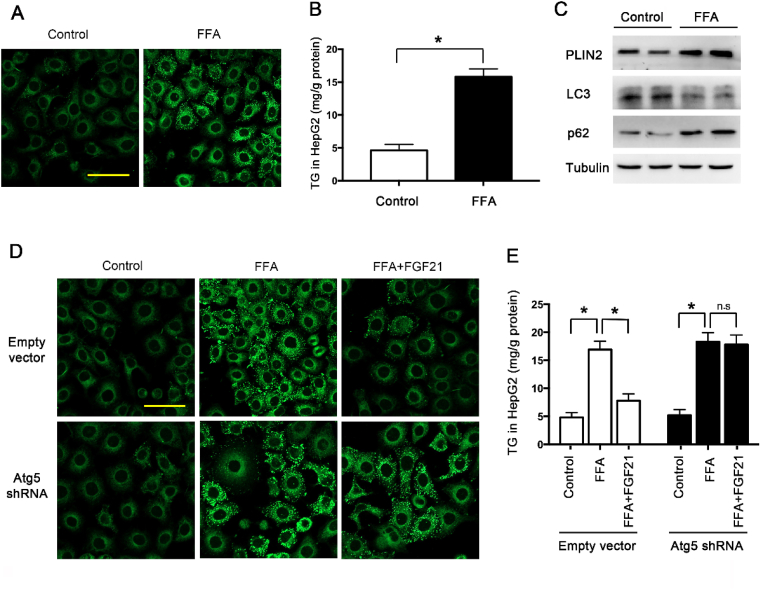
FGF21 attenuated LDs accumulation in FFA-treated HepG2 cells through an autophagy dependent way. HepG2 cells were treated with FFA (400 μM). (A) Representative confocal images of LDs by BODIPY 493/503 staining. Scale: 50 μm. (B) TG level in HepG2 cells. (C) Representative immunoblotting of PLIN2, LC3II/LC3I and p62 in HepG2 cells. HepG2 cells were pre-transfected with Atg5 shRNA or empty vector by lenti-virus, then treated with FFA (400 μM) with or without FGF21 (1 μg/ml). (D) Representative confocal images of LDs by BODIPY 493/503 staining. Scale: 50 μm. (E) TG level in HepG2 cells. Experiments were repeated three times. Data are shown as the means ± SEM. Significance was designated by asterisks with *p < 0.05.

The next question is how muscle-derived FGF21 regulates lipid metabolism in liver. Several studies have demonstrated that systemic administration of FGF21 can activate AMPK signaling and regulate energy expenditure [17]. From the data above, we have demonstrated exercise could significantly activate liver AMPK. To investigate whether FGF21 from muscle could induce lipophagy to attenuate lipid accumulation in liver through AMPK-dependent pathway, we employed the classical method of the mixture of oleate and palmitate to mimic dietary HFD to induce the lipid accumulation in the cultured heptocytes. The oleate and palmitate represents monounsaturated fatty acids and saturated fatty acids respectively. Studies have shown that the detrimental effects of FAs were mainly caused by saturated and monounsaturated fatty acids. The mixture of oleate and palmitate (2:1) has long been used in cellular model of NAFLD [18]. Studies have shown that in the short term FFA treatment induces autophagic protein degradation, but excess FFA treatment in the long term could impair autophagy. We treated HepG2 cells with FFA for 14 days to mimic the long-term treatment. FFA treatment indeed induced LDs accumulation shown by BODIPY staining (Fig. 4A), TG level (Fig. 4B) and PLIN2 level (Fig. 4C) in HepG2 cells. Furthermore, FFA treatment induced the decrease of LC3II/LC3I and the increase of p62 (Fig. 4C), indicating the inhibition of autophagy. BODIPY staining data (Fig. 4D) showed that FGF21 significantly decreased the FFA-induced LDs accumulation. To understand the role of autophagy in the protective effects of FGF21, Atg5 shRNA was employed to knockdown Atg5 protein to impair autophagy. In the Atg5 knockdown cells, the protection of FGF21 on LDs accumulation was almost abolished. This was further confirmed by the TG level in HepG2 cells (Fig. 4E). These results demonstrated that FGF21 treatment attenuated the LDs accumulation in an autophagy dependent way.

**Fig. 5:**
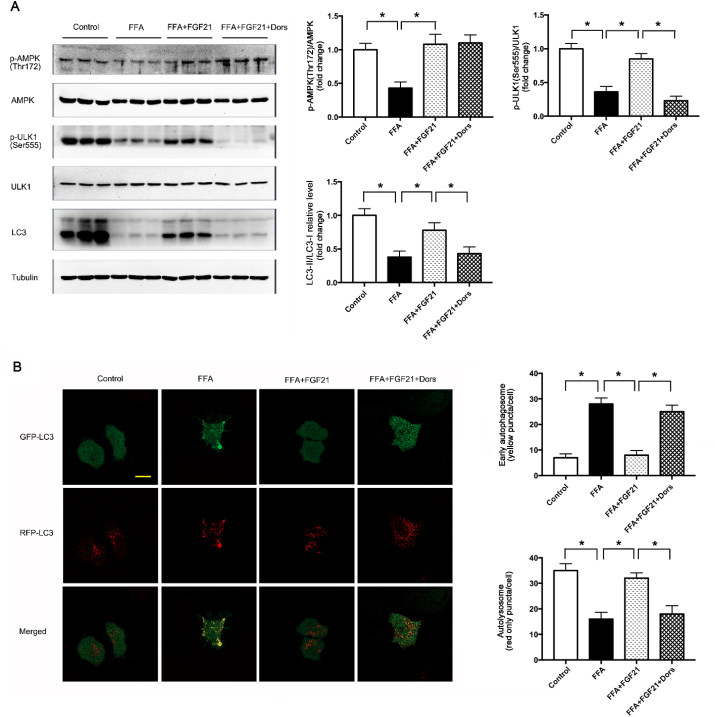
FGF21 increased lipophagy through AMPK-dependent pathway in FFA-treated HepG2 cells. HepG2 cells were treated with FFA (400 μM), FGF21 (1 μg/ml) as well as AMPK inhibitor-Dorsomorphin (1 μmol/L). (A) Representative of *p*-AMPK (Thr172)/AMPK total, *p*-ULK1 (Ser555)/ULK1 total and LC3-II/LC3-I immunoblotting in HepG2 cells treated with FFA, FGF21 and Dorsomorphin, and the quantified data. (B) Representative confocal images of HepG2 cells expressing GFP-RFP-LC3 and quantitation of early autophagosome puncta and autolysosome puncta following FFA, FGF21 and Dorsomorphin treatment. Yellow showed co-localization of GFP and RFP, indicating early autophagosomes. Red only showed autolysosomes, Scale: 20 μm. Experiments were repeated three times. Data are shown as the means ± SEM. Significance was designated by asterisks with *p < 0.05. (For interpretation of the references to colour in this figure legend, the reader is referred to the Web version of this article.)

To investigate whether FGF21 could induce autophagy to suppress lipid accumulation through AMPK-dependent pathway. We treated the FFA-challenged cells with FGF21 in the presence or absence of AMPK inhibitor (Dorsomorphin). Indeed, FFA treatment reduced the phosphorylations of AMPK (Thr172) as well as ULK1 (Ser555), and suppressed the autophagy shown by lower LC3II/LC3I protein level. FGF21 treatment significantly activated AMPK and ULK1 by increasing the phosphorylations of AMPK (Thr172) and ULK1 (Ser555). However, the activations of AMPK and ULK1 induced by FGF21 treatment were completely blocked by AMPK inhibitor (Fig. 5A). These results demonstrated that, FGF21 promoted the autophagy in the FFA treated cells through AMPK-dependent pathway. To confirm this, FFA treated cells were transfected with a tandem fluorescent tagged RFP-GFP-LC3 adenovirus to examine the autophagy flux. GFP reporters lose their fluorescence in the acidic environment of the autolysosome, whereas RFP is relatively stable. Thus, an increase in the ratio of red to green signal is a good indication of autophagic flux. The yellow signal represents early autophagosome while the red only signal represents autolysosome. The rising yellow signal with the declining red only signal suggests autophagic flux may be blocked. Our data showed that FFA treatment significantly blocked the autophagy flux, illustrated by more early autophagosome (yellow puncta on colocalization) with less autolysosome (red only puncta), whereas FGF21 treatment showed less early autophagosome with more autolysosome, and FGF21 effects were blocked by AMPK inhibitor (Fig. 5B). These results demonstrated FGF21 reversed FFA-mediated suppression of autophagy through activating AMPK pathway.

**Fig. 6:**
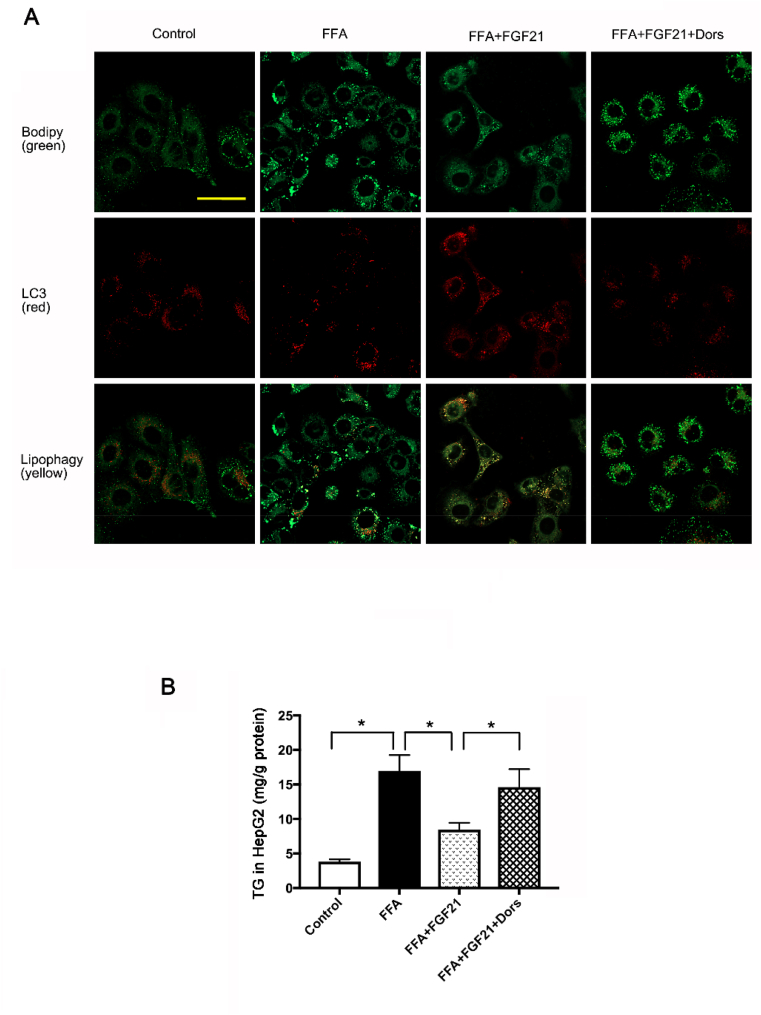
FGF21 increased the lipophagy and reduced the lipid accumulation in FFA-treated HepG2 cells. HepG2 cells were treated with FFA (400 μM), FGF21 (1 μg/ml) as well as AMPK inhibitor-Dorsomorphin (1 μmol/L). (A) Representative confocal images of colocalization of LDs and autophagosomes by BODIPY 493/503 staining in GFP-LC3 protein expressed HepG2 cells. Scale: 50 μm. (B) TG level in HepG2 cells treated with FFA, FGF21 and Dorsomorphin. Experiments were repeated three times. Data are shown as the means ± SEM. Significance was designated by asterisks with *p < 0.05.

As a specific type of autophagy, lipophagy is one of the major mechanisms to clear the lipid in the cells. To address the question whether lipophagy was also promoted by FGF21 in an AMPK dependent manner, we performed a colocalization study between the autophagosomal marker LC3 and LDs. Consistent with the data shown previously, autophagic LDs degradation was dramatically induced by the FGF21 treatment and AMPK inhibitor abolished this effect (Fig. 6A). Together, these data suggested that FGF21 could induce lipophagy through AMPK pathway under the FFA treatment condition, which appears to be the primary mechanism for FGF21 to reduce LDs accumulation in the cells.

To evaluate the role of lipophagy induced by FGF21 in LDs accumulation, LDs were stained with BODIPY. As shown in Fig. 6A, FGF21 effectively cleared the excessive LDs load induced by FFA treatment, while this effect was suppressed by AMPK inhibitor (Fig. 6A). Consistent with this result, FGF21 also reduced the TG level in the FFA treated cells, which could be effectively blocked by AMPK inhibitor (Fig. 6B). All these suggested FGF21 could induce lipophagy to suppress lipid accumulation in an AMPK dependent manner. This might be the crucial mechanism for improving NAFLD by exercise.

### 3.5 Exercise and dietary intervention ameliorated the HFD-induced liver aging

**Fig. 7:**
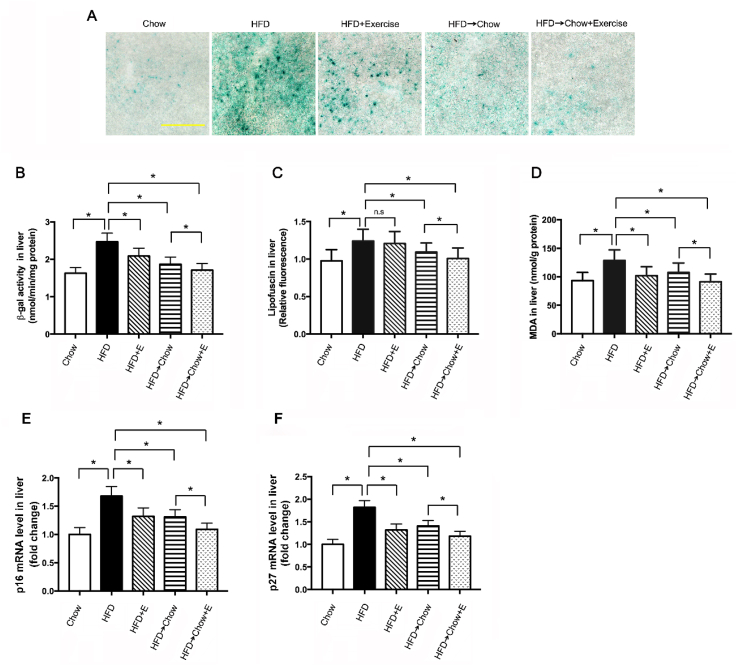
Exercise and dietary intervention both ameliorated the liver aging caused by HFD. (A) Representative SA-β-gal staining, Scale: 200 μm, and (B) SA-β-gal activity in the liver from rats on HFD with exercise and dietary intervention. (C) Liver lipofuscin and (D) MDA contents in the liver from rats on HFD with exercise and dietary intervention. (E) p16 and (F) p27 mRNA levels in liver from rats on HFD with exercise and dietary intervention. Data are shown as the means ± SEM, n = 8 per group. Significance was designated by asterisks with *p < 0.05.

Hepatic fat accumulation in NAFLD can trigger oxidative stress in the liver, as the excessive supply of lipids to the mitochondria would reduce β-oxidation and increase production of reactive oxygen species (ROS) [34]. Oxidative stress is regarded as the main cause of aging. So, we speculated that HFD could induce liver aging. SA-β-gal is the well-established marker to evaluate the aging [35]. In the animal study, we found that SA-β-gal activity increased significantly in the HFD group. Exercise and dietary intervention significantly inhibited HFD-induced increase of SA-β-gal activity (Fig. 7A–B). Next, we measured the products of oxidative injury, lipofuscin and MDA. As expected, the contents of lipofuscin and MDA all increased in HFD group, which were blocked significantly by exercise and dietary intervention (Fig. 7C–D). Cell cycle arrest is another hallmark in aging and increased CDKIs such as p16 and p27 are often used as the aging markers [36,37]. Consistently, HFD increased the levels of p16 and p27, on which exercise and dietary intervention showed significant inhibition (Fig. 7E–F). All above suggested exercise and dietary intervention could ameliorate the HFD-induced liver aging, and combination of these two treatments was more effective.

### 3.6 FGF21 ameliorated cell senescence through inducing AMPK dependent-autophagic pathway

**Fig. 8:**
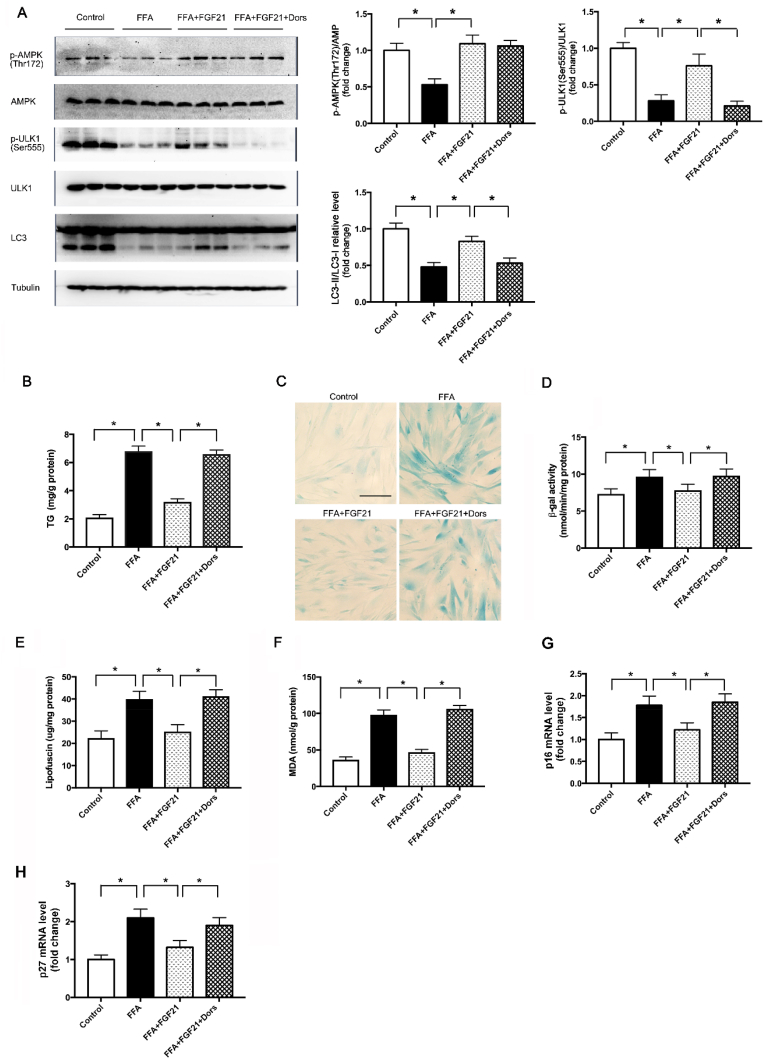
FGF21 ameliorated the cell senescence through AMPK dependent autophagic pathway in FFA-treated WI-38 cells. WI-38 cells were treated with FFA (400 μM), FGF21 (1 μg/ml) as well as AMPK inhibitor-Dorsomorphin (1 μmol/L). (A) Representative of *p*-AMPK (Thr172)/AMPK total, *p*-ULK1 (Ser555)/ULK1 total and LC3-II/LC3-I immunoblotting in WI-38 cells and quantified data. (B) TG level in WI-38 cells. (C) Representative SA-β-gal staining (Scale: 50 μm), (D) SA-β-gal activity, (E) lipofuscin and (F) MDA contents in WI-38 cells. (G) p16 and (H) p27 mRNA levels in WI-38 cells. Experiments were repeated three times. Data are shown as the means ± SEM. Significance was designated by asterisks with *p < 0.05.

As tumor cell line, such as HepG2, was not suitable for aging study, we employed WI-38 cell, a normal human fetal lung fibroblast cell line in the cell senescence study. We treated WI-38 cells with FFA and also successfully induced LDs accumulation in the cells (see Supplementary Fig. 2). To evaluate the role of AMPK-dependent autophagic pathway in aging, we tested the effect of FGF21 with or without AMPK inhibitor in FFA-treated WI-38 cells. The results showed FGF21 reversed the reduction of AMPK and ULK1 phosphorylation as well as LC3 II/LC3I level caused by FFA treatment, and then decreased the TG level in the cells in an AMPK-dependent manner (Fig. 8A and B), which was consistent with our finding that FGF21 attenuated LDs accumulation in an AMPK-dependent autophagic pathway.

To evaluate the effect of LDs accumulation on aging, we further detected aging marker SA-β-gal, oxidative stress markers lipofuscin and MDA as well as the cell cycle arrest markers p16 and p27. FFA treatment dramatically increased aging related markers while FGF21 treatment significantly reduced these changes, same as the role of exercise in rats on HFD. Again, these effects of FGF21 on cell senescence were abrogated by AMPK inhibitor (Fig. 8C–H). All these data indicated that lipid accumulation accelerated cell senescence, and FGF21 could ameliorate this process via inducing AMPK-dependent autophagic pathway.

## 4 Discussion

With the prevalence of obesity and metabolic disease, NAFLD has become a growing global phenomenon. Changes in life style such as exercise and dietary intervention have been established as the effective treatments for NAFLD, while the underlying mechanism is still unclear. In the present study, we showed exercise and dietary intervention improved the NAFLD by increasing lipophagy through different pathways. Exercise activated the AMPK/ULK1 pathway to increase the lipophagy directly, while dietary intervention reversed the lipophagy inhibition caused by HFD via Akt/mTOR/ULK1 pathway. Moreover, combination of these two treatments were more effective to improve NAFLD. FGF21, an important metabolic regulator, is reported to be involved in glucose homeostasis, lipid metabolism, and energy balance [38,39]. Our results showed that exercise increased the production of FGF21 in the muscle, which remotely upregulated the lipophagy in the liver to reduce the lipid accumulation through activating AMPK pathway. Additionally, exercise and dietary intervention also improved the liver aging caused by excessive lipid accumulation.

Many studies have indicated that autophagy was involved in the lipid metabolism, and identified a dysregulation in the autophagic response in the liver following chronic HFD exposure [4,40,41]. As a specific autophagy, lipophagy selectively removes excess lipids, and maintains lipid homeostasis in the cells. Hence promoting lipophagy might be an effective strategy to ameliorate liver steatosis [6]. In the present study, our results showed both exercise and dietary intervention effectively upregulated autophagy and lipophagy through simulating AMPK/ULK1 pathway as well as suppressing the overactivation of Akt/mTOR/ULK1 pathway respectively. Akt/mTOR is a nutrient sensing pathway, and our results added to this by demonstrating that this pathway was activated strongly by HFD, while it could not be reversed by exercise. mTOR was the upstream kinase for ULK1 at Ser 757, and Akt/mTOR/ULK1 pathway was involved in the nutrient-induced autophagy [30,42]. Work from Møller et al. [29] had found that ULK1 phosphorylation at Ser757 in the muscle was not affected by physical exercise in human. Interestingly, in our present study, although exercise had no obvious effect on Akt/mTOR, the phosphorylation of ULK1 at Ser757 was slightly decreased in the liver. This suggested that even in the muscle, the phosphorylation of ULK1 at Ser757 was not affected by exercise, while in the liver, other effectors from the muscle during exercise might regulate the phosphorylation of ULK1 at Ser757. Notably exercise induced significant increase of AMPK Thr172 and ULK1 Ser555 phosphorylation, and then promoted autophagy and lipophagy in the liver. Thus, our results demonstrated that during exercise, muscle could pass autophagy and lipophagy initiation signal to the liver, and the activation of AMPK/ULK1 pathway in liver was the potential underlying mechanism. Moreover, the combination of exercise and dietary intervention was more effective on the induction of lipophagy. However, in a human clinical trial, combining exercise with energy restriction had no further changes in body composition [43]. In another study, a low-fat, high-carbohydrate diet combined with increased physical exercise resulted in greatest weight loss and reduced total cholesterol and low-density lipoprotein-cholesterol [44]. Although controversies existed as to whether the combination of dietary intervention with exercise could improve body composition or body weight than exercise or CR alone, our results indicated that combination of exercise and dietary intervention could be more beneficial to alleviate NAFLD. This study supported dietary intervention in conjunction with physical exercise to control NAFLD.

Previous study has shown that exercise stimulates autophagy in liver [45], but the underlying mechanism remains unknown. FGF21 is a member of FGF family isolated from liver in 2000 [46], and liver is considered to be a major source of circulating FGF21 [47]. In 2008, FGF21 was found to be expressed not only in liver, but also in skeletal muscle [48]. It is accepted as a true myokine with endocrine actions as its co-receptor β-Klotho is not expressed in skeletal muscle [49]. Under normal physiological conditions, the basal level of FGF21 in liver is low; however, during fasting and starvation, both hepatic and serum FGF21 levels are elevated dramatically, indicating FGF21 is inducible [47]. Consistent with this concept, several studies demonstrated that increases in muscle contraction such as chronic exercise also upregulate muscular FGF21 production [50]. Now FGF21 has been accepted as an exercise-induced myokine and its circulating level is elevated in response to submaximal exercise [[51], [52], [53], [54]], and increased FGF21 secreted from muscle has been regarded as a protective stress response [48,55,56]. Recent two studies by employing the skeletal muscle specific FUNDC1-knockout mice or skeletal muscle specific TSC1-knockout mice respectively, demonstrated that muscle-derived FGF21 significantly affected the FGF21 level in serum [57,58]. In the present study, our data demonstrated that exercise could significantly increase FGF21 level in serum, which confirmed the previous findings [[51], [52], [53], [54]]. By comparing the changes of FGF21 levels in liver and muscle, we found that muscle was the primary source of FGF21 production response to exercise.

As the largest metabolic and secretory organ, crosstalk between muscle and other organs, such as liver, adipose, brain, has been discussed [57,[59], [60], [61]]. Exercise-released myokines have been identified to confer the benefit of metabolic improvement [62,63]. One study showed the increased *p*-AMPK level in liver after exercise [64], suggesting the role of AMPK pathway in mediating the effects of exercise-induced myokines. To understand the function of FGF21 in fatty liver, we investigated the supplementation of exogenous FGF21 on the lipid accumulation in the fat-loaded hepatocytes. We found that FGF21 could significantly reduce the TG level and even the lipid droplets accumulation in the hepatocytes. Consistent with the data from in vivo, FFA treatment suppressed the AMPK/ULK1 pathway as well as autophagy and lipophagy, which was ameliorated by exogenous FGF21 supplementation. Specific AMPK inhibitor completely blocked the effects of FGF21 supplementation, demonstrating that FGF21 induced autophagy and lipophagy in an AMPK-dependent manner. It was reported that exercise could induce irisin expression in the muscle and secretion into circulation to exert its effects on ameliorating hepatic glucose/lipid metabolism [65,66]. However, the results from the numerous studies on the Irisin response to the exercise training are contradictory. In most of the studies, acute exercise increased the Irisin level in the plasma, while chronic exercise had no effect on the plasma level of Irisin [67]. This suggested that the type of the exercise training may affect the Irisin expression in the muscle. In our study, chronic exercise for 8 weeks did not change the Irisin level in the circulation (Supplementary Fig. 1).

Aging has been shown to be a risk factor of NAFLD [[68], [69], [70], [71]]. With aging, the liver undergoes anatomical and functional changes that are associated with significant impairment of many hepatic metabolic and detoxification activities [72]. Moreover, the progressively increased production of ROS during aging contributes to the lipid accumulation, particularly cholesterol, in the liver of aged mice [73]. The oxidative stress has been demonstrated to be one of the most important mechanism in the pathogenesis of NAFLD [74]. On the other hand, excessive lipid accumulation would impair mitochondrial function, which further increases ROS production and aggravates oxidative stress [75]. Oxidative stress is considered as the primary cause of the general aging as well as those diseases associated with aging, such as metabolic and heart disease [76]. There may be a positive feedback between aging and NAFLD. However, the role of aging on NAFLD has already been reported extensively in the literature, while few studies to date have tackled the question on if and how lipid accumulation affects the aging process. Our results in the present study showed lipid accumulation could accelerate aging in the liver, in which oxidative stress may be involved. Exercise and dietary intervention both attenuated the liver aging illustrated by the aging related markers (SA-β-gal staining, SA-β-gal activity, lipofuscin, MDA, p16 and p27).

As we have demonstrated above that exercise-induced FGF21 could improve lipid accumulation by inducing lipophagy in an AMPK-dependent pathway, to explore the role of improved lipid accumulation by FGF21 in aging, we treated FFA-challenged WI-38 cells with FGF21 as well as AMPK inhibitor. Consistent with the results in liver, lipid accumulation accelerated cell senescence. FGF21 increased autophagy, reduced lipid accumulation and improved FFA-induced cell senescence. AMPK inhibitor could effectively block these protective effects from FGF21. These findings supported the notion that the lipid metabolic disorder accelerated organs senescence. In both hepatocytes and fibroblasts, FGF21 reduced the lipid accumulation by inducing lipophagy to slow down the aging. A recent review summarized the beneficial effects of exercise on NAFLD, in which reducing intrahepatic fat content, increasing β-oxidation of fatty acids, inducing hepato-protective autophagy, overexpressing peroxisome proliferator-activated receptor-γ (PPAR-γ), attenuating hepatocyte inflammation and apoptosis, as well as decreasing oxidative stress had been proposed to be possible mechanisms of exercise effect on NAFLD [77]. Based on the results in our study, FGF21 induced AMPK-dependent lipophagy may be another mechanism of exercise improving NAFLD as well as liver aging. It will be interesting to test if FGF21 has the similar function in other organs such as heart and vascular.

**Fig. 9:**
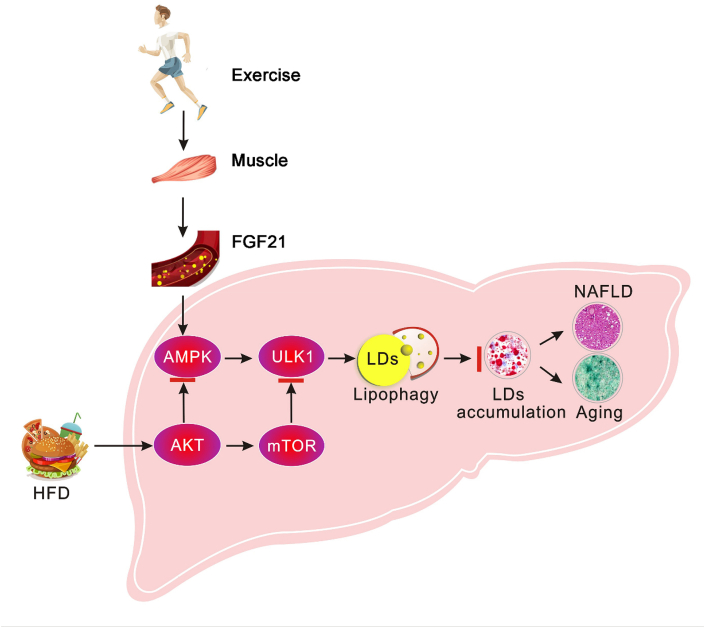
Schematic representation of lipophagy induced by exercise and dietary intervention through different pathways in liver to ameliorate HFD-induced NAFLD and liver aging.

Currently, exercise and dietary intervention are considered as clinically effective therapies to control NAFLD. For the first time, our findings demonstrate that exercise and dietary intervention up-regulate lipophagy in the liver by different molecular pathways, and consequently improving NAFLD and liver aging caused by HFD. Combined application of these two therapies has enhanced efficacy since they function through different signaling pathways. Furthermore, we find exercise stimulates muscle to produce FGF21 and secrete to the circulation, which remotely regulates liver lipophagy to improve NAFLD and liver aging. A diagrammatic outline of the mechanism in this study is shown in Fig. 9. This new mechanism could provide rationale for combined therapy for the lipid metabolic diseases.

## Funding sources

This work was supported by 10.13039/501100001809National Natural Science Foundation of China (31670863, 81270417, 81573127, 81470537) and 10.13039/501100007128Natural Science Foundation of Shaanxi Province (2016JZ027, 2019JM-130). This work was also supported by the Ministry of Education Key Lab of Hazard Assessment and Control in Special Operational Environment, Shaanxi Key Laboratory of Free Radical Biology and Medicine, and State Key Laboratory of Cancer Biology.

## Declaration of competing interest

The authors declared no conflict of interest.
