# Supplementary material for: Exercise and dietary intervention ameliorate high-fat diet-induced NAFLD and liver aging by inducing lipophagy
Source: Redox Biol. 2020 Jul 7;36:101635. doi: 10.1016/j.redox.2020.101635 (PMC7365984; doi:10.1016/j.redox.2020.101635)
Supplement: Multimedia component 1 [file mmc1.docx]

**Supplementary Data**

**Supplementary Figure 1:** Exercise did not induce the secretion of the Irisin to the serum. The Irisin level in the serum of rats was determined by ELISA. Data are shown as the means ± SEM, n = 8 per group. Significance was designated by asterisks with *p < 0.05.


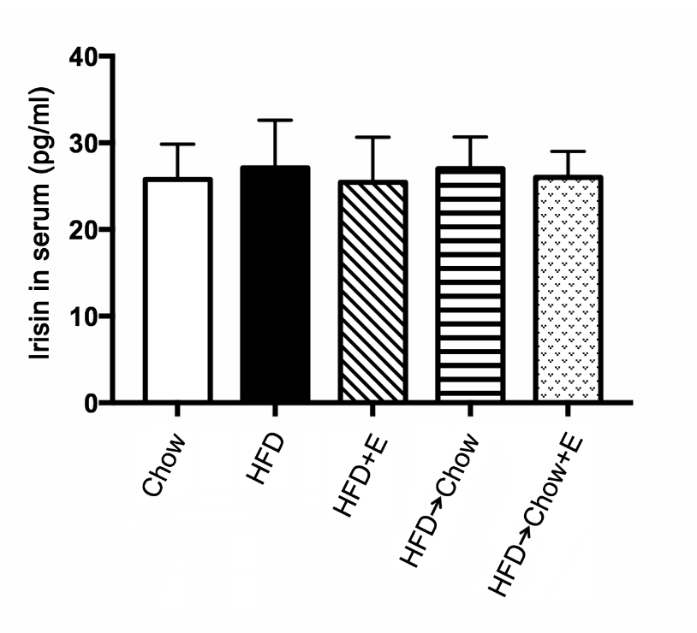


**Supplementary Figure 2:** FFA treatment induced lipid accumulation in WI-38 cells. WI-38 cells were treated with FFA (400 μM). (A) Representative confocal images of LDs by BODIPY 493/503 staining. Scale: 50 μm. (B) TG level in WI-38 cells. (C) Representative immunoblotting of PLIN2 in WI-38 cells and quantified data. Experiments were repeated three times. Data are shown as the means ± SEM. Significance was designated by asterisks with *p < 0.05.


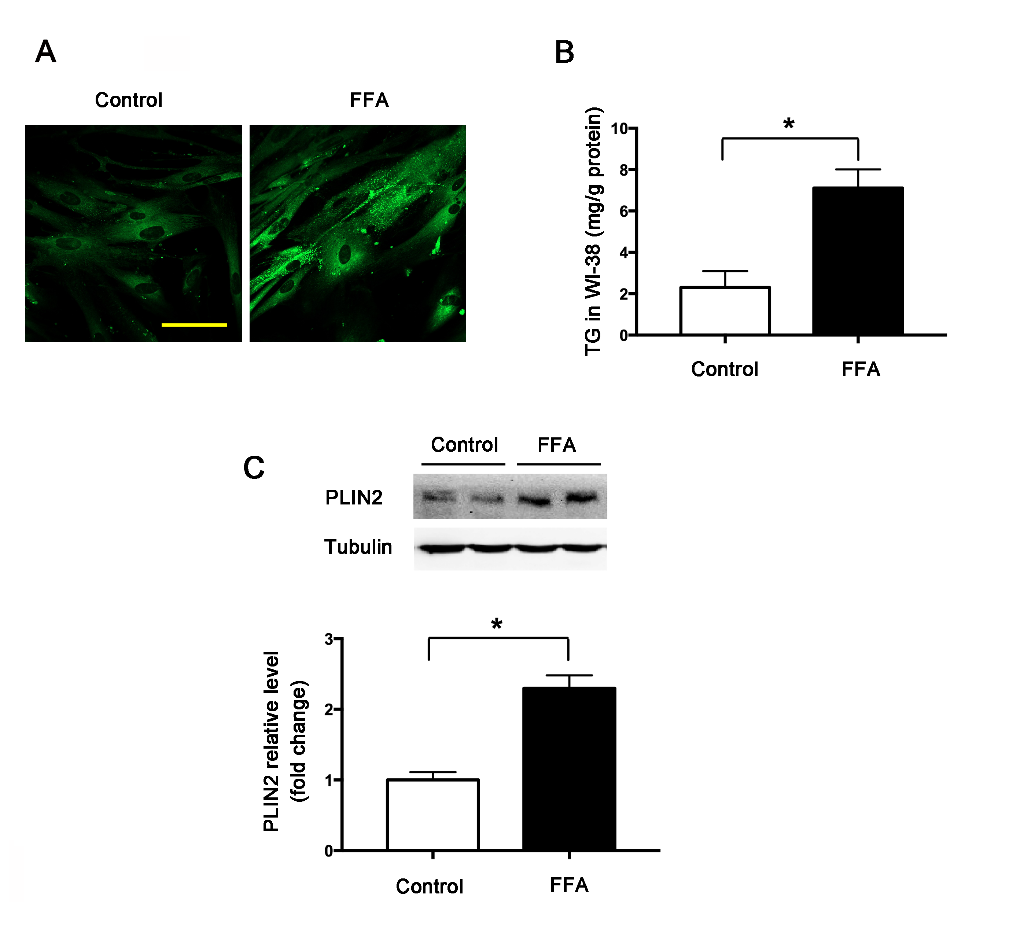


**Supplementary Table 1:** Outline of the pre-exercise training protocol for adaptation used to train rats.

| Day of training | Speed (m/min) | Time (min) |
| --- | --- | --- |
| Day 1 | 5 | 30 |
| Day 2 | 7 | 30 |
| Day 3 | 10 | 45 |
| Day 4 | 15 | 60 |
| Day 5 | 20 | 60 |
